# Time trends in prostate cancer screening in Swiss primary care (2010 to 2017) – A retrospective study

**DOI:** 10.1371/journal.pone.0217879

**Published:** 2019-06-13

**Authors:** Stefan Zechmann, Stefania Di Gangi, Vladimir Kaplan, Rahel Meier, Thomas Rosemann, Fabio Valeri, Oliver Senn, on behalf of the FIRE Study Group

**Affiliations:** 1 Institute of Primary Care, University of Zurich, University Hospital Zurich, Zurich, Switzerland; 2 Department of Internal Medicine, Hospital Muri, Muri, Switzerland; Icahn School of Medicine at Mount Sinai, UNITED STATES

## Abstract

**Introduction:**

Following years of controversy regarding screening for prostate cancer using prostate-specific antigen, evidence evolves towards a more restrained and preference-based use. This study reports the impact of landmark trials and updated recommendations on the incidence rate of prostate cancer screening by Swiss general practitioners.

**Methods:**

We performed a retrospective analysis of primary care data, separated in 3 time periods based on dates of publications of important prostate-specific antigen screening recommendations. 1: 2010-mid 2012 including 2 updates; 2: mid 2012-mid 2014 including a Smarter Medicine recommendation; 3: mid-2014—mid-2017 maintenance period. Period 2 including the Smarter Medicine recommendation was defined as reference period. We further assessed the influence of patient’s age and the number of prostate-specific-antigen (PSA) tests, by the patient and within each time period, on the mean PSA concentration. Uni- and multivariable analyses were used as needed.

**Results:**

36,800 men aged 55 to 75 years were included. 14.6% had ≥ 2 chronic conditions, 11.7% had ≥ 1 prostate-specific antigen test, (mean 2.60 ng/ml [SD 12.3]). 113,921 patient-years were covered. Data derived from 221 general practitioners, 33.5% of GP were women, mean age was 49.4 years (SD 10.0), 67.9% used prostate-specific antigen testing. Adjusted incidence rate-ratio (95%-CI) dropped significantly over time periods: Reference Period 2: incidence rate-ratio 1.00; Period 1: incidence rate-ratio 1.74 (1.59–1.90); Period 3: incidence rate-ratio 0.61 (0.56–0.67). A higher number of chronic conditions and a patient age between 60–69 years were significantly associated with higher screening rate. Increasing numbers of PSA testing per patient, as well as increasing age, were independently and significantly associated with an increase in the PSA value.

**Conclusion:**

Swiss general practitioners adapted screening behavior as early as evidence of a limited health benefit evolved, while using a risk-adapted approach whenever performing multiple testing. Updated recommendations might have helped to maintain this decrease. Further recommendations and campaigns should aimed at older patients with multimorbidity, to sustain a further decline in prostate-specific antigen screening practices.

## Introduction

Screening for prostate cancer (PC) using prostate-specific antigen (PSA) has been controversial for many years. As evidence evolved, recommendations changed towards a more restrained and preference-based use.

In 2009 two randomized controlled landmark trials on screening for PC, the Prostate, Lung, Colorectal, and Ovarian Cancer Screening (PLCO) and the European Randomized Study of Screening for Prostate Cancer (ERSPC) study provided different results for PC screening using PSA in terms of the balance between benefits and harms, causing uncertainty among some clinicians [1, 2]. These two studies and many others that followed resulted in an adaptation of PC screening recommendations by health authorities. In Switzerland, the presumably most recognized recommendations and campaigns were: November 2011 the Swiss Medical Board (SMB) recommended against PSA-based PC screening among asymptomatic men without risk factors [3]. May 2012 the United States Preventive Services Task Force (USPSTF) recommended against PSA-based PC screening in any men in the general population [4, 5]. May 2014 the Swiss Society of General Internal Medicine issued a shortlist of low-value health care activities leading to the “Swiss Smarter Medicine” (SSM) campaign building on the growing international “Choosing Wisely” campaign [6, 7]. The SSM campaign recommended against PSA testing without a discussion of the risks and benefits and offering PSA-based screening to men <75 years.

Studies on PC screening that investigated the impact of changing screening recommendations on clinical practice over time reported varying results and focused on the 2012 USPSTF update [8–14]. In Switzerland, the latest data analyzing PC screening trends date back to 2013 [15, 16] and evaluate the impact of the 2011 SMB recommendation. For Switzerland, data on PSA testing trends in primary care evaluating the impact of the two landmark trials (PLCO, ERSPC) are lacking.

The aim of this study was to analyze the incidence rate of PSA testing for PC screening among men in Swiss primary care since 2010, focusing on a time period following the publication of the PLCO and ERSPC trials and other screening recommendations (e.g. SMB 2011, USPSTF 2012, SSM 2014). Furthermore, we investigated patients and general practitioners (GP) characteristics for an association with PSA testing for PC screening.

## Materials and methods

For this retrospective longitudinal study we calculated the incidence rates of PSA testing in Swiss primary care male patients from 2010 to 2017.

### FIRE database

We used the primary care research database FIRE (Family medicine ICPC-Research using Electronic medical records) between January 2010 and June 2017.

The FIRE database contains standardized data extracted fully anonymized from routine electronic medical records on patient-physician encounters in participating practices. Any Swiss GP with electronic medical records available is welcome to participate in the FIRE project. Details of FIRE were reported previously [17–22].

### Patient population

**Fig 1:**
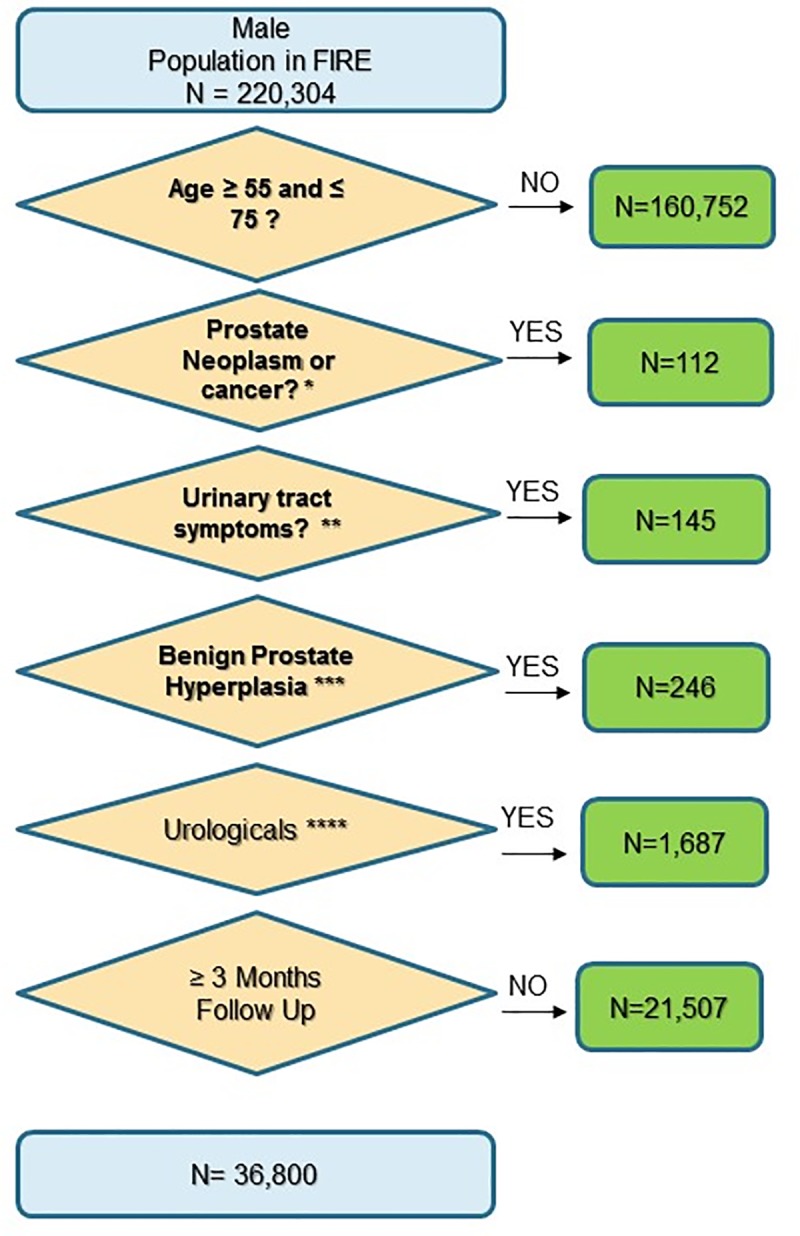
Flow chart. Fig 1 shows the inclusion and exclusion criteria: *prostate neoplasm/cancer based on ICPC-2 codes: [Y77-Y79], ** urinary tract symptoms based on ICPC-2 codes: [U01-U08], *** benign prostate hyperplasia based on ICPC-2 code [Y85], **** urologic medications based on ATC code [G04].

All male patients between the ages of 55 to 75 years were eligible. Details on the criteria for inclusion and exclusion of patients are described in Fig 1.

### Time periods, patient and GP characteristics

We defined three periods of interest: Period 1 = [01/01/2010-30/06/2012] including the SMB and USPSTF updates; Period 2 = [01/07/2012-30/06/2014] including the SSM shortlist; Period 3 = [01/07/2014-30/06/2017] without any recommendation updates. As the Smarter Medicine recommendations (Period 2) were building on the growing international Choosing Wisely campaign we defined period 2 as the reference period.

Patient characteristics: age, chronic conditions (as defined by Lamers et al. and O`Halloran et al. as previously shown by Zellweger et al.), value and number of PSA measurements and number of consultations [20, 23, 24]. GP characteristics: year of birth, gender, number of patients, number of consultations, type of practice and practice location [25].

### Statistical analysis

All descriptive data are presented as means and standard deviations (SD) for continuous and as numbers (N) and percentages (%) for categorical variables.

To control for a potential chance of a (wrong) “survival bias” based on the fact that the FIRE database does not include information on death, we considered expected lifetime at each patients`last consultation date, based on the Federal Statistical Office life table [26]. When the patients`expected lifetime did not go beyond the study end, the follow up ended at the computed death date, otherwise, we used the end of the study, 30/06/2017.

The crude incidence rates (IR) of PSA testing for PC screening, as well as the adjusted incidence rate ratios (IRR), were calculated as follows:

a) For all eligible patients with and without stratification for patients`age group, number of chronic conditions, and time periods. Furthermore stratified crude PSA testing rates were calculated according to GP characteristics.

b) To assess the independent effect of time periods, patient and GP characteristics on PSA testing rates we set up a multilevel Poisson model with individual encounters as first level clustering variable (repeated measurements due to multiple encounters), with patient characteristics as second level clustering variable (age, comorbidity, time period), and GP characteristics as third level clustering variable (year of birth, gender, practice location).

We performed univariable and multivariable analyses using a GLM (Poisson) mixed model with nested random effects (patients in GP), specified as follows:

IR=numberofPSA/patient‐years(PY)∼[Fixedeffect(s)(X)+randomeffectsofintercept=GP/patients]

where X = (period of time + patient age group + comorbidity + practice location).

In univariable analysis, every effect (X) was considered separately in a single model. In multivariable analysis, instead, all the above effects were considered together.

We further assessed the influence of patient’s age and the number of PSA tests, by the patient and within each time period, on the mean PSA concentration. Therefore, the value of the PSA screening test, in logarithmic scale, was modeled through a linear mixed model, univariable and multivariable, with nested random effects at two levels: patients and providers. The logarithmic transformation was used to make the distribution normal and it was already used in previous research [26]. We considered, as independent predictors, the total number of PSA screening tests made in the same time period, and the age of the patient computed at the date of examination. Both predictors were defined as categorical variables.

In summary, we had:

Log(PSAvalue)∼Fixedeffect(s)(X)+randomeffectsofintercept=providers/patients]

where X = (total number of PSA in the period + patient age at examination).

Since we use the logarithmic transformation, the coefficients of the categorical predictors represented the differences, in the expected geometric means, of the log of PSA value between the categories of each predictor.

For all regression models, the acceptable type 1 error was set at p≤0.05. R (Version 3.3.2) was used for analyses and data management [27].

## Results

### Patient population

36,800 men could be included. Reasons for exclusion are detailed in Fig 1.

The mean age of patients was 61.2 (SD 7.2) years. 5,384 (14.6%) had ≥ two chronic conditions, 4,299 (11.7%) had ≥ one PSA test (overall 6,112; 2% of all consultations). Mean PSA value was 2.6 ng/ml (SD 12.3). Mean observation time, or length of follow-up, was 3.1 (SD 2.3) years.

82 practices with 221 GPs were included. 74 (33.5%) were women. Mean age was 49.4 (SD 10.0) years. 77.8% worked in group practices located in urban areas (48.4%). 67.9% used PSA testing. Details are shown in Tables 1 and 2.

**Table 1:** Patient characteristics.

Characteristics	
Number of patients	36,800
Age {year}	
Mean (SD)	61.2 (7.2)
≥ 2 Chronic conditions	5,384 (14.6%)
n (%)	
Mean (SD)	0.6 (1.1)
**PSA** {ng/ml}	
Number of patients with PSA	4,299 (11.7%)
n (%)	
Number of PSA (total)	6,112
Number of PSA per patient	1.4
PSA value	
Mean (SD)	2.60 (12.3)
Patient-years	113,921
Length of follow-up	
Mean (SD)	3.1 (2.3)
Number of PSA per consultation	
Mean (SD)	0.02 (0.07)

Table 1 shows the characteristics used for uni- and multivariable analyses only. Abbreviations: SD = standard deviation, n = number of observations.

**Table 2:** GP characteristics.

Characteristics	
Number of GPs	221
Number of GPs working in group practices	
n (%)	172 (77.8%)
Number of practices	82
Age {year}	
Mean (SD)	49.4 (10.0)
Gender {female}	
n (%)	74 (33.5%)
Number of male patients per GP*	
Mean (SD)	166 (185)
PSA	
Number of GPs testing	
n (%)	150 (67.9%)
Number of PSA per GP	
Mean (SD)	28 (58)
Consultations per day	
Mean (SD)	19.7 (12.6)
Number of PSA per year	
Mean (SD)	11.9 (14.7)
Location of practice **	
n (%)	
Urban area	107 (48.4%)
Sub-urban area	56 (25.3%)
Rural area	18 (8.1%)
Other areas	35 (15.8%)

Table 2 shows GP characteristics with reference to the patients included in the study. * only patients included in the study were considered.

** for one practice (0.4%) data on location of practice was missing. Abbreviations: GP = general practitioner, SD = standard deviation, n = number of observations.

### The incidence rate of PSA testing and determinants

**Fig 2:**
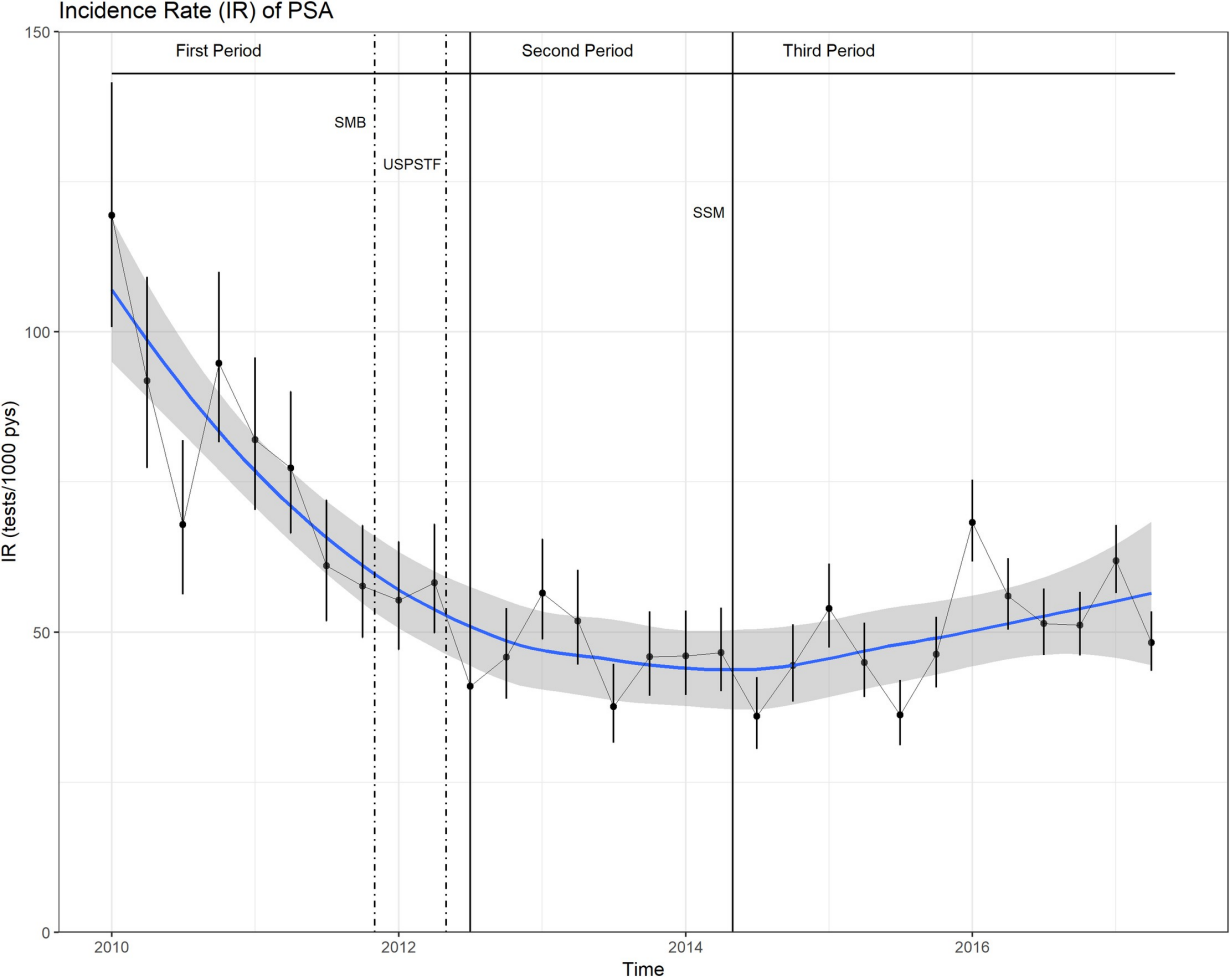
Incidence rate of PSA (observed). Fig 2 shows the observed Incidence rates of PSA screening (tests per 1000 patient-years). Points represent incidence rates calculated by quarters. Error bars represent 95% confidence intervals. The blue line represents the smoothing and the gray area represents the 95% confidence interval band. Solid vertical lines identify the three time periods. Dotted lines identify the date of important publications. Abbreviations: IR = incidence rate, pys = patient-years, SMB = Swiss Medical Board recommendation, USPSTF = United States Preventive Services Task Force recommendation, SSM = Swiss Smarter Medicine campaign.

The observed IR decreased significantly during the study period, in particular during the first time period (01/01/2010-30/06/2012) and stabilized during the second (01/07/2012-30/06/2014) and third (01/07/2014-30/06/2017) time period (Fig 2).

When considering observation period 2 as the reference period, the univariable IRR for PSA testing in the first period was significantly higher: IRR 1.56; 95%-CI (1.45, 1.69) and in the third time period significantly lower: IRR 0.73; 95%-CI (0.68, 0.78). Incidence rates of PSA testing remained significantly different between time periods when controlled for patient and GP characteristics (Table 3).

**Table 3:** PSA screening incidence rate and its determinants.

	Patient-years	IR (tests/1000 pys)	IRR Univariable (95% CI)	p-value	IRR Multivariable (95% CI)	p-value
All (N = 36,800)	113,921	54				
**Patient characteristics**						
**Age group**						
55−59 years	46,588	45	1	-	1	-
60−64 years	27,485	60	1.30 (1.23,1.42)	< 0.001	1.26 (1.18, 1.36)	< 0.001
65−69 years	23,567	65	1.40 (1.29, 1.50)	< 0.001	1.31 (1.22, 1.42)	< 0.001
70−75 years	16,280	51	1.10 (1.03, 1.24)	0.009	1.09 (0.99, 1.19)	0.067
**Chronic conditions**						
0–1	88,625	50	1	-	1	-
> = 2	25,296	67	1.60 (1.47, 1.70)	< 0.001	1.68 (1.56, 1.81)	< 0.001
**Time period**						
[01/01/2010-30/06/2012]	20,321	72	1.56 (1.45, 1.69)	< 0.001	1.67 (1.54, 1.81)	< 0.001
[01/07/2012-30/06/2014]	26,670	46	1	-	1	-
[01/07/2014-30/06/2017]	66,930	51	0.73 (0.68, 0.78)	< 0.001	0.73 (0.68, 0.79)	< 0.001
**GP characteristics**						
**Year of birth**						
1940–1959	61,409	38	1	-		
1960–1969	21,346	63	1.00 (0.49, 2.05)	0.994		
1970–1989	14,428	76	1.40 (0.73, 2.68)	0.307		
**Gender (missing = 1)**						
male	105,721	54	1.16 (0.64, 2.11)	0.615		
female	8,019	41	1	-		
**Location of practice**						
Urban area	34,487	39	1	-	1	-
Sub-urban area	44,423	64	2.00 (1.05, 3.80)	0.035	1.57 (0.77, 3.19)	0.212
Rural area	15,766	82	2.31 (0.88, 6.06)	0.088	1.97 (0.68, 5.74)	0.212
Other areas	18,640	35	1.03 (0.49, 2.17)	0.936	1.03 (0.45, 2.34)	0.942

Table 3 shows the incidence rate of PSA screening stratified according to time periods, patients and GP characteristics. Abbreviations: IR = Incidence rate, N = number of patients, CI = Confidence Interval, GP = general practitioner, IRR = incidence rate ratio, pys = patient-years.

Increasing patient age to the age group 65–69 years and at least two chronic conditions were independent determinants of a higher PSA testing incidence in the multivariable model (Table 3). In the age group 70–75 the incidence rate of PSA testing did not significantly differ from the younger age group (55–59 years, which was the reference age group used). A sensitivity analysis comparing the incidence rates of PSA testing among those aged 50–54 (IR 28 tests/1000 pys (patient-years); total: 24,594 pys) as well as in those aged older than 75 years (IR 25 tests/1000 pys; total: 31,589 pys) to incidence rates of patients among the recommended age group for PSA screening yielded significant lower incidence rates.

Increasing numbers of PSA testing per patient, as well as increasing age, were independently and significantly associated with an increase in the PSA value (Table 4). In fact, after controlling for patient age, the PSA value, when 5 PSA tests were done, was (exp(1.23)-1)*100 = 242% higher compared with the PSA value when only 1 PSA test was made. Analogously, after controlling for the number of PSA tests, when the patient age was 70–75, the PSA value was (exp(0.205)-1)*100 = 23% higher compared with the PSA value of a patient aged 55–59.

**Table 4:** Regression analysis of PSA level (on log-scale).

Variable		Univariable		Multivariable	
Number of PSA tests per patient	Log(PSA) value ng/ml Mean (SD)	Estimate (95% CI)	P-value	Estimate (95% CI)	P-value
1 (N = 4,384)	0.10 (1.09)	-	-	-	-
2 (N = 616)	0.21 (1.36)	0.11 (0.04, 0.18)	0.002	0.10 (0.03, 0.17)	0.005
3 (N = 119)	0.44 (1.80)	0.31 (0.19, 0.44)	< 0.001	0.29 (0.17, 0.41)	< 0.001
4 (N = 25)	1.10 (1.43)	0.39 (0.11, 0.66)	0.006	0.38 (0.11, 0.65)	0.007
5 (N = 3)	1.39 (0.79)	1.27 (0.08, 2.47)	0.037	1.23 (0.04, 2.43)	0.042
6 (N = 4)	1.71 (1.18)	0.36 (-0.24, 0.97)	0.237	0.34 (-0.26, 0.95)	0.267
**Age at PSA testing**					
55–59 (N = 1333)	0.00 (0.95)	-	-	-	-
60–64 (N = 1144)	0.17 (1.08)	0.14 (0.07, 0.21)	< 0.001	0.14 (0.07, 0.21)	< 0.001
65–69 (N = 1034)	0.26 (1.28)	0.22 (0.14, 0.30)	< 0.001	0.20 (0.12, 0.28)	< 0.001
70–75 (N = 788)	0.25 (1.56)	0.22 (0.13, 0.31)	< 0.001	0.20 (0.12, 0.29)	< 0.001

Table 4 shows associations between log PSA level with the number of tests and patient’s age. Results of univariable and multivariable models are showed as estimates and 95% confidence intervals. Period of time and patient age group were both considered in univariable and multivariable analysis. Observed means and standard deviations of the natural logarithm of PSA, by the number of tests and patient’s age, are also reported. Abbreviations: N = number of patients, CI = Confidence Interval, SD = Standard Deviation.

## Discussion

Swiss GPs reduced PSA screening once evidence to be more cautious evolved. The biggest reduction in PSA screening was observed within 3 years after the publications of the landmark trials (PLCO and ERSPC) and before the updated screening recommendations of various authorities (e.g. SMB, USPSTF, and SSM). Patients`older age and a higher number of chronic conditions were independently associated with higher screening rates.

Concerning the influence of recommendations, previous authors interpreted their data both ways. Either as having no or minimal effect on PSA screening [12, 15, 28, 29] or leading to a decline [8–11, 13, 30–32]. Based on a similar cohort of asymptomatic men, Ong et al. were the only one previously reporting a decline before the release of the 2012 USPSTF recommendation [33].

For Switzerland Eichler et al. were the only ones trying to evaluate the impact of SMB recommendations on PSA testing from 2005 until 2013. Direct comparison with previous studies is difficult due to differences concerning methodology, especially different datasets (Eichler et al. used general claims data rather than data derived from GPs outpatients only) and different time periods used. Similar to Guessous et al. who used data from five waves (from 1992–2012) of the population-based Swiss Health Interview Survey [16], did not report a general decline starting from 2010/11. Concerning the impact of recommendations, they estimated a statistically significant reduction in tests immediately after the intervention but were not able to address its long term effect [15].

We believe that the early decline we found, especially in 2010 and 2011, reflects increased awareness of the limitations of PC screening after the publication of the 2 large randomized, controlled trials in 2009 (PLCO in the United States and the ERSPC in Europe]), which showed limited health benefit. Though it is not clear what really triggered this trend, one might hypothesize that the controversial discussion in the lay media, as well as the pro-cons debate in the scientific literature, resulted in increased awareness and uncertainty, on both the GPs`and the patients`side [34–41]. Thus, published evidence resulted in a decrease in PSA screening in clinical practice before health authorities issued updated recommendations. While Fig 2 visually depicts the impression that numbers increased again during the third time period, the multivariable analysis yielded further reduction during this period, strengthening the value of updated recommendations rather for “maintenance” than actually changing GPs`behavior. These results fit previous data on how doctors react to guidelines and where their decisions derive from, namely from “mindlines” rather than guidelines as first described by Gabbay et al. more than a decade ago [42, 43].

During the study period of seven years, 12% of our patients had at least one PSA test. In this respect, our results fit in the lower cumulative testing rate in comparison to previous studies reporting proportions between 10 and 70% of all men in industrialized countries [14, 16, 28, 34, 44, 45]. For Switzerland Altwegg et al. reported in the 2012 Swiss health report that 25% of all men between 50 and 74 years of age had an PSA test within the last year in 2007 and Guessous et al. reported that 42.4% of all men specified in an questionnaire they had received a PSA test in 2012 [16, 44]. These results are best explained by different methodologies, time spans and data source used [46, 47].

For an “age-adjusted population” our study population was healthier (e.g. has fewer co-morbidities) therefore making them eligible for PSA screening. We found that multiple chronic conditions were significantly associated with a higher screening rate. This might be explained by the fact that patients with multimorbidity have higher consultation rate as reported by Bowser et al. (odds for screening rise by 2.36–6.78). [48, 49]. This finding suggests an optimization of testing in this already high-risk group of patients and highlights the importance of further addressing expected benefits as well as harms of screening.

Data concerning the impact of GPs`age and gender on PSA screening are scant. Eisinger et al. reported a higher frequency of systematic recommendation for breast cancer screening among female GPs. Lofter et al. reported a trend toward reduced screening among older physicians for other types of cancer [37, 50], likely a generation change or trend of the time. Therefore, we expected a higher rate for prostate cancer screening among older GPs in general, and male GPs in particular [37]. However, we did not find a statistically significant association between screening and gender or age …

Concerning association with rural-urban divide, previous studies yielded diverse results. Some reported increased PSA screening in urban [16, 51], others in rural areas [52, 53], most likely reflecting regional as well as national differences. For Switzerland, we cannot report any significant association either way.

PSA value increased significantly in relation to the number of PSA measurements in the same patient as well as patients`age. This result persisted when controlled for the cluster GP, thus indicating GP used a risk-adapted approach whenever they performed multiple testing [54, 55].

### Strengths

Our study has important strengths.

First, the relatively large number of patients, as well as the long study period of 7 years are certainly strengths of this study. Even more, as most studies published on this topic were published immediately after or within the first 2 or 3 years after the publication of the recommendations, which did not allow them to address the long term effect of these. Many authors claimed that a longer time frame was needed to interpret the actual impact of guidelines on PSA screening.

Second, multilevel modeling to control for confounding e.g. means controlled for repeated measurements and GP clustering strengthens our results.

Third, the composite of ICPC-2 and ATC coding does improve the quality of the morbidity indicators in electronic health records like ours, as previously shown by Busato et al. [17].

### Limitations

However, the study also had certain limitations.

First, we did not have accurate information to address the clinical reason for PSA testing. Our eligibility criteria addressed an age range where PSA testing might be a result of a shared decision-making process between the GP and the patient. Patients suffering from urinary tract symptoms and disorders defined by ICPC-2 diagnostic codes or taking urologic medications according to the ATC medication classification have been excluded, therefore limiting the analysis to asymptomatic men representing a screening population. The fact that only 20% of the patients had two or more chronic conditions contrasts the higher prevalence of multimorbidity that can be expected in a primary care study population in the same age range [19] assuming a relatively healthy population making them eligible for PSA screening. In addition, the mean measured PSA value was within the normal range (< 4.0 ng/mL) and testing incidence in younger (<55 years) and older men (>75 years) outside the screening age was lower, further indicating that the results apply to a screening setting.

When interpreting our results one has to keep in mind, that we only included men that had an encounter with their GP during the study period. The Swiss health statistics 2012 reported that approximately 60–80% of all Swiss men, depending to their age, had at least one GP encounter within the last 12 month[44]. Similar Guessous et al. reported that 74% of their patients had a GP contact within the last 12 months *[16].* Therefore we estimate that at most 20% of all men in the general population did not have a GP encounter during the 9 years and were not included in this study.

Third, we cannot exclude the possibility that PSA testing rates were already decreasing in Switzerland prior to the publication of the PLCO and ERSPC trials. An additional period prior to the publication would be of great interest, unfortunately was our database was found in 2009, therefore we do not have sufficient data for an additional time period.

## Conclusion

Swiss GPs adapted their PC screening behavior as early as evidence of a limited health benefit evolved while using a risk-adapted approach whenever performing multiple testing. Thus, PSA testing rate significantly decreased from 2010 to 2012, while subsequent campaigns and recommendations had only a minor impact on further testing incidence rates. Older age and a higher number of chronic conditions of the patients were associated with higher screening rates. Further recommendations and campaigns should be aimed at these subgroups, to maintain and further decline PC screening practices to individual patients giving them the opportunity to discuss the potential benefits and harms of screening [56].

## Supporting information

S1 Table S1 Table shows a minimal anonymized data set necessary to replicate our study findings. and includes an additional data set description. (XLSX)
